# A gut commensal *Serratia marcescens* inhibits dengue virus infection via prodigiosin-induced autophagy in *Aedes albopictus*

**DOI:** 10.1080/21505594.2026.2721757

**Published:** 2026-09-08

**Authors:** Yijia Huang, Hongbo Li, Jinying Ding, Yihan Zhu, Nan Wang, Jiawei Zhang, Haojie Wang, Xiao Feng, Haodong Xu, Wenquan Liu, Shaohui Liang

**Affiliations:** a Department of Parasitology, School of Basic Medical Sciences, Wenzhou Medical University, Wenzhou, Zhejiang, China; b Department of Pathogenic Biology, School of Basic Medical Sciences, Soochow University, Suzhou, Jiangsu, China

**Keywords:** Dengue virus, *Aedes albopictus*, gut microbiota, *Serratia marcescens*, Prodigiosin, autophagy

## Abstract

The mosquito gut microbiota plays a pivotal role in regulating arbovirus transmission, yet the specific antiviral metabolites produced by native symbiotic bacteria and their underlying mechanisms remain poorly understood. In this study, we isolated a natural gut symbiotic bacterium, *Serratia marcescens* strain WZ1, from field-caught *Aedes albopictus* in Wenzhou, China, and demonstrated its potent ability to inhibit dengue virus (DENV) infection. Through integrated metabolomic analysis, we identified the red pigment prodigiosin (PG) as a functional antiviral metabolite secreted by this strain. PG treatment suppressed DENV infection in mosquito cells in a dose- and time-dependent manner and significantly reduced DENV2 RNA levels in adult *Ae. albopictus* midguts. Mechanistic investigations revealed that PG preferentially localizes to the endoplasmic reticulum (ER), where it induces ER stress and upregulates the chaperone protein GRP78. This process subsequently activates a complete autophagic flux, as evidenced by enhanced conversion of Atg8-I to Atg8-II, increased autophagosome formation, and elevated lysosomal activity. Crucially, we further demonstrated that PG facilitates the convergence of autophagosomes and lysosomes, culminating in the colocalization of DENV with lysosomal compartments and subsequent viral clearance. Both genetic knockdown of the autophagy gene *Atg8* and pharmacological inhibition of ER stress substantially attenuated PG-mediated viral suppression, confirming the functional link between PG-induced ER stress, autophagy activation, and viral clearance. Our findings elucidate a novel mechanism by which a native mosquito gut symbiont metabolite restricts arboviral infection through activation of the host ER stress–autophagy pathway, providing a mechanistic basis and candidate leads for future transmission-blocking studies.

## Introduction

Dengue virus (DENV), a member of the *Flaviviridae* family, poses a significant global public health threat, with an estimated 100–400 million infections annually [1]. As the primary vectors responsible for DENV transmission, *Aedes aegypti* and *Aedes albopictus* mosquitoes have seen their geographic ranges expand, escalating the risk of epidemic outbreaks [2]. The current prevention and control systems face dual challenges: on one hand, there remains a lack of specific antiviral drugs and universal effective vaccines against DENV [3,4]; on the other hand, current vector control strategies, mainly relying on insecticide-based interventions, are increasingly challenged by the emergence of insecticide resistance and the failure to sustainably control mosquito populations [2]. Therefore, the development of novel, sustainable, and eco-friendly strategies to interrupt DENV transmission is an urgent priority in vector biology.

The mosquito midgut constitutes the primary entry portal and a major physiological barrier for arboviruses. The midgut epithelium not only facilitates nutrient absorption but also mounts robust defensive responses against invading pathogens, encompassing physical integrity and innate immune mechanisms [5,6]. Furthermore, accumulating evidence underscores the role of the gut microbiota in modulating susceptibility to several human pathogens, including DENV, Zika virus (ZIKV), and *Plasmodium* parasites [7,8]. Complex communities of symbiotic bacteria influence viral infection through multiple mechanisms: regulating basal immune activity, altering the integrity of physical barriers, and secreting metabolites or enzymes with direct antiviral or proviral activity [9–11]. For instance, *Enterobacter hormaechei*-derived sphingosine conferred resistance to ZIKV/DENV infection in *Aedes* mosquitoes [12], while *Chromobacterium* species inhibits DENV infection by degrading the E protein via aminopeptidase [13]. Despite the profound influence of bacterial symbionts, the identification of specific natural mosquito-derived bacterial strains with consistent anti-DENV activity and particularly the characterization of their active metabolites, remain largely unexplored.

Prodigiosin (PG), a striking red pigment belonging to the bacterial prodiginine family, is notably produced by *Serratia marcescens*, a common member of the mosquito gut microbiota [14]. This microbial metabolite has attracted increasing attention due to its broad-spectrum biological activities, including antibacterial, antifungal, anticancer, and immunosuppressive properties [15]. Recent studies further indicate its potential as an antiviral agent, showing efficacy against viruses such as herpes simplex virus (HSV) and *Bombyx mori* nucleopolyhedrovirus (BmNPV) via suppression of viral gene transcription and disruption of host cell survival pathways [16,17]. Nevertheless, whether PG exerts any antiviral effect on arthropod-borne viruses such as DENV, and the potential molecular mechanisms underlying these effects in mosquito cells, remain entirely unknown.

In this study, we isolated and identified *S. marcescens* strain WZ1 from the midgut of field-caught *Ae. albopictus*. The secreted metabolite PG demonstrated significant anti-DENV activity in both mosquito cells and adult midguts. We further revealed that PG induces endoplasmic reticulum (ER) stress, which in turn activates a complete autophagic flux, culminating in the degradation of virions through enhanced autolysosomal formation. Our findings revealed that the metabolite of *S. marcescens* strain WZ1 exploits host autophagy machinery to combat DENV infection in *Ae. albopictus*, highlighting its potential as a novel strategy for blocking DENV transmission from a mosquito-borne perspective.

## Materials and methods

### Cells, virus, and mosquitoes

*Aedes albopictus* C6/36 cells (ATCC® CRL-1660™) and Aag2 cells were maintained in Leibovitz’s L-15 medium (Gibco) and Schneider’s Drosophila medium (Gibco) supplemented with 10% fetal bovine serum (FBS) and antibiotics at 28°C in a 5% CO_2_ atmosphere. The DENV2 New Guinea C (NGC) strain was propagated in C6/36 cells as previously described [18]; the cell culture supernatant was harvested 6 days post-infection, aliquoted, and stored at −80°C for subsequent infections.

The Foshan strain of *Ae. albopictus* was reared and blood-fed as described previously [19]. Briefly, adult mosquitoes were maintained under conditions at 28°C and 80% relative humidity, with a 12-h light/dark cycle. Adults were bred in cages and provided with a 10% sucrose solution via cotton wicks. Female mosquitoes were starved for 15 h prior to receiving a blood meal. The artificial blood meal consisted of sterile defibrinated sheep blood mixed 1:1 (v/v) with DENV2 viral stock (3 × 10^6^ PFU/mL), which was pre-warmed at 37°C for 30 minutes. Mosquitoes were fed for 2 h using a membrane-based feeder apparatus and maintained at 37°C. Fully engorged females were then transferred to clean cages and subsequently maintained on a 10% sucrose solution.

### Isolation, identification, and cultivation of gut commensal bacteria

Female *Ae. albopictus* mosquitoes, caught from the field in Wenzhou, were anesthetized at 4°C. The surface of the mosquitoes was sterilized with 75% ethanol and subsequently washed twice with PBS. Midguts were aseptically dissected, homogenized, and subjected to ten-fold serial dilutions. The diluted homogenates were plated onto antibiotic-free LB agar plates and incubated at 28°C. Single colonies were inoculated into LB liquid medium for expansion, and then identified by 16S rRNA gene sequencing and comparison [12]. All identified bacterial strains were preserved in 50% glycerol at −80°C for storage.

### Cytotoxicity assay

The identified bacterial strains were inoculated at a 1:100 ratio into LB liquid medium and cultured for 12 h at 28°C with shaking at 250 rpm. The supernatant was collected by centrifugation at 11,000 × g and filtered through a 0.22-μm membrane filter. A volume of 10 μL of the filtered supernatant was added to C6/36 cells seeded in 96-well plates, followed by incubation for 48 h. Cytotoxicity was assessed using a CCK-8 kit (Beyotime, C0038) by measuring the absorbance at 450 nm. The same procedure was applied to evaluate the cytotoxicity of PG and 4-phenylbutyric acid (4-PBA).

### Anti-DENV activity assay of bacterial supernatant and prodigiosin *in vitro*

C6/36 cells (1 × 10^6^) were seeded into 12-well plates and cultured for 12 h at 28°C. The cells were then infected with DENV2 NGC strain at an MOI 0.8 for 2 h at 37°C. After infection, the inoculum was replaced with L-15 maintenance medium containing 2% FBS, supplemented with either 10 μL of bacterial culture supernatant or PG at final concentrations of 25 nM or 75 nM. The cells were co-cultured with these treatments for 24 h or 48 h at 28°C.

To determine the major phase during which PG exerts its antiviral effect, C6/36 cells were pretreated with 75 nM PG for 2 h before DENV2 infection. The PG-containing medium was then removed, and cells were washed before DENV2 adsorption. For the post-entry treatment, cells were first infected with DENV2 for 2 h, after which the viral inoculum was removed and replaced with maintenance medium containing 75 nM PG. Total RNA and protein were extracted from the harvested cells using TRIzol™ reagent (Invitrogen, 15596018). The RNA and protein expression levels of DENV2 *NS1* and other target genes were analyzed by RT-qPCR and western blot, respectively. To validate the antiviral effect of PG in an additional mosquito cell line, Aag2 cells were infected with DENV2 at an MOI of 0.8 for 2 h. After removal of the viral inoculum, cells were cultured in maintenance medium containing 75 nM PG and harvested at 24 h for western blot analysis of DENV2 NS1 protein.

### RNA extraction and viral quantification by RT-qPCR

Total RNA was extracted using TRIzol™ Reagent (Invitrogen, 15596018) according to the manufacturer’s protocol. cDNA was synthesized from the extracted RNA using HiScript II Q RT SuperMix (Vazyme, R223). Quantitative PCR was subsequently performed with ChamQ SYBR qPCR Master Mix (Vazyme, Q311) on a QuantStudio 6 Flex Real-Time PCR System (Applied Biosystems). The thermocycling conditions were as follows: initial denaturation at 95°C for 10 min, followed by 40 cycles of denaturation at 95°C for 15 sec and annealing/extension at 60°C for 60 sec. Viral RNA levels were quantified by RT-qPCR targeting the DENV2 NS1 genomic region and normalized to the mRNA level of the mosquito ribosomal protein S7 (*Rps7*) gene [20]. The sequences of primers used for qPCR amplification are listed in Table S1.

### Protein preparation and western blot analysis

Protein was extracted from cell lysates using the TRIzol™ Reagent. After removing the aqueous phase containing RNA, proteins were precipitated with isopropanol, and dissolved in 1% SDS for denaturation. Protein concentration was determined using a BCA protein assay kit (Beyotime, P0010S). Total protein samples were separated by SDS-PAGE and transferred onto polyvinylidene fluoride (PVDF) membranes (Millipore, IPVH00010). The membranes were blocked with 5% skim milk in Tris-buffered saline containing 0.1% Tween-20 (TBS-T) and then incubated with the desired primary antibodies at 1:2000 dilution: NS1 (GeneTex, GTX124280), mTOR (Abcam, ab32028), phospho-mTOR (Abcam, ab109268), Atg8 (MBL Life Science, PM037), SQSTM1/p62 (GeneTex, GTX100685), and β-actin (QiHe Biotechnology, 040010003). After washing, membranes were incubated with horseradish peroxidase (HRP)-conjugated goat anti-rabbit IgG secondary antibody (Bioshark, BL003A) at a 1:10000 dilution for 1 h at room temperature. Signal detection was performed using a SuperPico ECL Chemiluminescence Kit (Vazyme, E422). Protein band intensities were quantified by densitometry using ImageJ, and target protein expression levels were normalized to those of β-actin.

### Phylogenetic analysis

To determine the phylogenetic position of *Serratia* sp. WZ1, its 16S rRNA gene sequence was used as a query in BLAST searches. A total of 14 closely related type strains belonging to the genus *Serratia* and 5 highly similar strains from the family *Enterobacteriaceae* were selected as references. Multiple sequence alignment was performed using the Clustal-W algorithm implemented in MEGA 11. A phylogenetic tree was constructed using the Maximum Likelihood method with 1000 bootstrap replications to assess node support.

### Gut microbiota depletion and bacterial recolonization in mosquitoes

Forty anesthetized female mosquitoes per group were starved for 15 h and then fed a 10% sucrose solution containing 20 U/mL penicillin and 20 µg/mL streptomycin for 5 days to clear gut microbiota [21]. For bacterial recolonization, *S. marcescens* WZ1 was inoculated at 1:100 into antibiotic-free LB broth and cultured for 12 h at 28°C with shaking at 250 rpm. Bacterial cells were harvested by centrifugation at 5,000 × g, and resuspended in 10% sucrose solution. Prior to recolonization, mosquitoes were starved for 24 h to metabolize residual antibiotics. The bacterial suspension was provided with sugar meal for 3 days, after which mosquitoes were starved for 15 h and subsequently infected with DENV2 via blood meal.

To assess clearance efficiency, midguts were homogenized, serially diluted, and plated for colony counting. To reduce the influence of inter-individual variation in mosquito gut bacterial loads, surviving mosquitoes from each group were ranked according to their absolute gut bacterial abundance after antibiotic treatment or bacterial recolonization, and then sequentially divided into three pooled samples. Each treatment pool was compared with the corresponding control pool, and CFU data are presented as relative values. In parallel, total bacterial DNA was extracted from individual mosquito midguts using the TIANamp Bacteria DNA Kit (TIANGEN, DP302), and bacterial abundance was quantified via qPCR targeting the 16S rDNA of commensal bacteria or *S. marcescens*. Primer sequences for amplification are listed in Table S1.

### Assessment of anti-dengue activity of *S. marcescens* WZ1 and prodigiosin in *Ae. albopictus*

Female *Ae. albopictus* mosquitoes subjected to antibiotic treatment, recolonization of *S. marcescens* WZ1, or without treatment (negative control) were orally infected with DENV2 via blood meal. At 3 days post-infection, midguts were dissected and homogenized, followed by centrifugation at 12,000 × g for 5 min at 4°C, and the supernatants were used for RNA and protein extraction.

To evaluate the antiviral effect of PG *in vivo*, 5 μM PG was mixed 1:1 (v/v) with pre-warmed DENV2-defibrinated sheep blood and provided to mosquitoes via artificial membrane feeding. At 3 days post-infection, mosquito midguts were dissected and processed for RNA extraction. For RT-qPCR analysis, three dissected midguts were pooled as one sample for viral RNA quantification to improve detection reliability.

### Identification and comparison of metabolites from *S. marcescens* WZ1 by HPLC-MS/MS

The culture supernatant of *S. marcescens* WZ1 was filtered through a 0.22-μm membrane and analyzed using an Ultimate 3000 high-performance liquid chromatography system (Thermo) coupled with a TripleTOF 5600+ mass spectrometer (AB SCIEX). Raw MS data were preprocessed using MS-DIAL 4.70 (Data-independent MS/MS deconvolution for comprehensive metabolome analysis) software [22] and the extracted spectral peaks were matched against three public metabolomic databases: MassBank, ReSpect, and GNPS. Prodigiosin identified in the supernatant of *S. marcescens* WZ1 was further verified by comparing its mass-to-charge (m/z) ratio with that of a commercial prodigiosin standard (Abcam, ab144331).

### Immunofluorescence microscopy

Immunostaining and fluorescence microscopy were performed as previously described [23]. C6/36 cells were seeded on coverslips in 24-well plates and cultured for 12 h at 28°C. Cells were infected with DENV2 at an MOI 0.8 for 2 h, followed by replacement with medium containing PG and further incubation for 24 or 48 h. Cells were fixed with 4% (v/v) paraformaldehyde for 15 min and permeabilized with 0.1% Triton X-100 for 10 min. After blocking with 5% BSA for 1 h, samples were incubated overnight at 4°C with the following primary antibodies or probes: anti-NS1 (GeneTex, GTX124280), anti-Atg8 (MBL Life Science, PM037), anti-Calnexin (Affinity Biosciences, AF5362), and LysoTracker Green (Beyotime, C1047S). Cells were then incubated with an AF647-conjugated goat anti-rabbit IgG secondary antibody (Beyotime, A0468) for 1 h. Nuclei were stained with DAPI for 10 min, and coverslips were mounted using Antifade Mounting Medium (Beyotime, P0126) before imaging. Images were acquired using a Leica STELLARIS 5 confocal microscope. LysoTracker Green, intrinsic PG fluorescence, and AF647 signals were detected using the corresponding green, orange-red, and far-red channels, respectively. PG was excited at 561 nm, and its intrinsic fluorescence was collected at 570–630 nm. Over six random fields were selected for analysis; the mean fluorescence intensity (MFI) of target proteins was quantified, and Pearson correlation coefficient was calculated for colocalization analysis using ImageJ.

### siRNA transfection and inhibitor treatment

Autophagy was suppressed by knockdown of *Atg8* via RNA interference. Briefly, C6/36 cells were transfected with small interfering RNA (siRNA) using Lipofectamine 2000 transfection reagent (Thermo, 11668019) in Opti-MEM I Reduced-Serum Medium (Gibco, 31985070) according to the manufacturer’s instructions. Cells were harvested 24 h post-transfection, and knockdown efficiency was evaluated by RT-qPCR and western blot. The designed sequences of *Atg8* siRNA (si*Atg8*) are listed in Table S1.

ER stress was inhibited using the small-molecule inhibitor 4-phenylbutyric acid (4-PBA). C6/36 cells were pretreated with 1 mM 4-PBA for 2 h prior to DENV2 infection. After 2 h of viral adsorption, the medium was replaced with virus-free medium containing 4-PBA and PG, and the cells were further cultured for 48 h.

### Statistical analysis

Statistical analyses were performed using GraphPad Prism 9. Data are presented as mean ± SEM unless otherwise indicated. The statistical tests used for each experiment are indicated in the figure legends. For experiments performed in multiple independent repeats, data were normalized to the corresponding control group within each experiment when appropriate and then pooled for statistical analysis. For western blot quantification, target protein levels were first normalized to β-actin and then to the corresponding control group from the same experiment before pooling independent experiments. For immunofluorescence analysis, mean fluorescence intensity and Pearson correlation coefficient were quantified from randomly selected fields or cells using ImageJ, as indicated in the figure legends. For mosquito experiments, the unit of analysis was either individual mosquitoes or pooled mosquito samples, as specified in the corresponding figure legends. Unpaired t test was used for comparisons between two groups, and one-way or two-way ANOVA with multiple-comparison tests was used for comparisons among multiple groups. The numbers of biological replicates, independent experiments, and analyzed mosquitoes, fields, or cells are indicated in the figure legends.

## Results

### Intestinal *Serratia* from *Aedes albopictus* inhibits DENV infection

**Figure 1.**
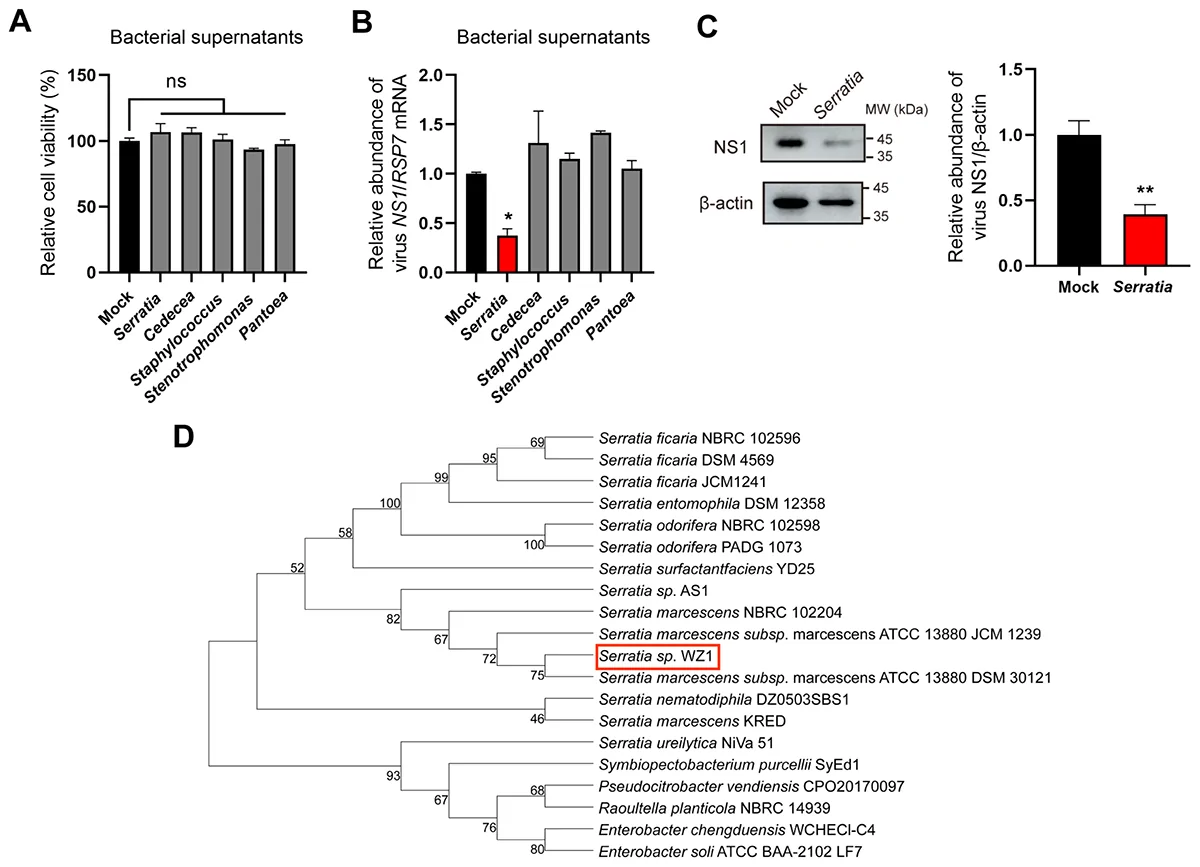
Identification of a gut commensal *Serratia* isolate from field-caught *Ae. albopictus* with anti-DENV activity. (A) Cytotoxicity of gut commensal bacteria. Five bacterial isolates from field-caught *Ae. albopictus* were identified by 16S rRNA gene sequencing as *Serratia, Cedecea*, *Staphylococcus, Stenotrophomonas*, and *Pantoea*. Cytotoxicity of bacterial culture supernatants toward C6/36 cells was assessed by CCK-8. (B, C) Evaluation of the anti-DENV effect of the commensal isolates. C6/36 cells infected with the DENV2 New Guinea C (NGC) strain were treated with supernatants from the five bacterial cultures for 48 h. Intracellular RNA levels of the DENV2 non-structural protein 1 (NS1) gene were measured by RT-qPCR (B). NS1 protein expression in the *Serratia* treatment group was analyzed by western blot (C). (D) Phylogenetic analysis of *Serratia* sp. WZ1. A maximum-likelihood phylogenetic tree was constructed using MEGA 11, including the antiviral *Serratia* sp. WZ1 isolate, 14 highly homologous strains of the genus *Serratia*, and 5 reference strains from the family *Enterobacteriaceae*. Data were representative or pooled results from three independent experiments and are shown as mean ± SEM. Ordinary one-way ANOVA with Dunnett’s multiple comparisons test (A and B) or unpaired t test (C) were used to determine the significance (*p < 0.05, **p < 0.01, ns, not significant).

Gut commensal bacteria have been implicated in regulating the infection of a variety of mosquito-borne pathogens, including DENV [24]. *Aedes albopictus* and *Aedes aegypti* serve as the primary vectors for DENV transmission. To identify resident microbes capable of modulating DENV infection under natural conditions, we isolated the commensal flora from the midgut of field-caught *Ae. albopictus* in Wenzhou, China. 16S rRNA sequencing identified five cultivable bacterial species including *Serratia*, *Cedecea*, *Staphylococcus*, *Stenotrophomonas*, and *Pantoea* (Figure 1).

To evaluate the antiviral potential of these isolates, cell-free supernatants from *in vitro* bacterial cultures were applied to DENV2-infected C6/36 cells. Cytotoxicity assays confirmed that these supernatants did not affect host cell viability (Figure 1(A)). Notably, only supernatants from *Serratia* strain significantly suppressed DENV2 replication, reducing intracellular viral non-structural protein 1 (NS1) RNA levels by 62.5% (*p* < 0.05, Figure 1(B)). This reduction was corroborated at the protein level, as shown by decreased NS1 expression in *Serratia*-treated cells (Figure 1). Phylogenetic analysis classified the antiviral isolate as *Serratia marcescens*, which we designated *Serratia marcescens* WZ1 (Figure 1). Together, these results identify a native gut-associated *S. marcescens* strain with significant inhibitory activity against DENV.

### *S. marcescens* WZ1 colonization in the midgut enhances the resistance of *Ae. albopictus* to dengue infection

**Figure 2.**
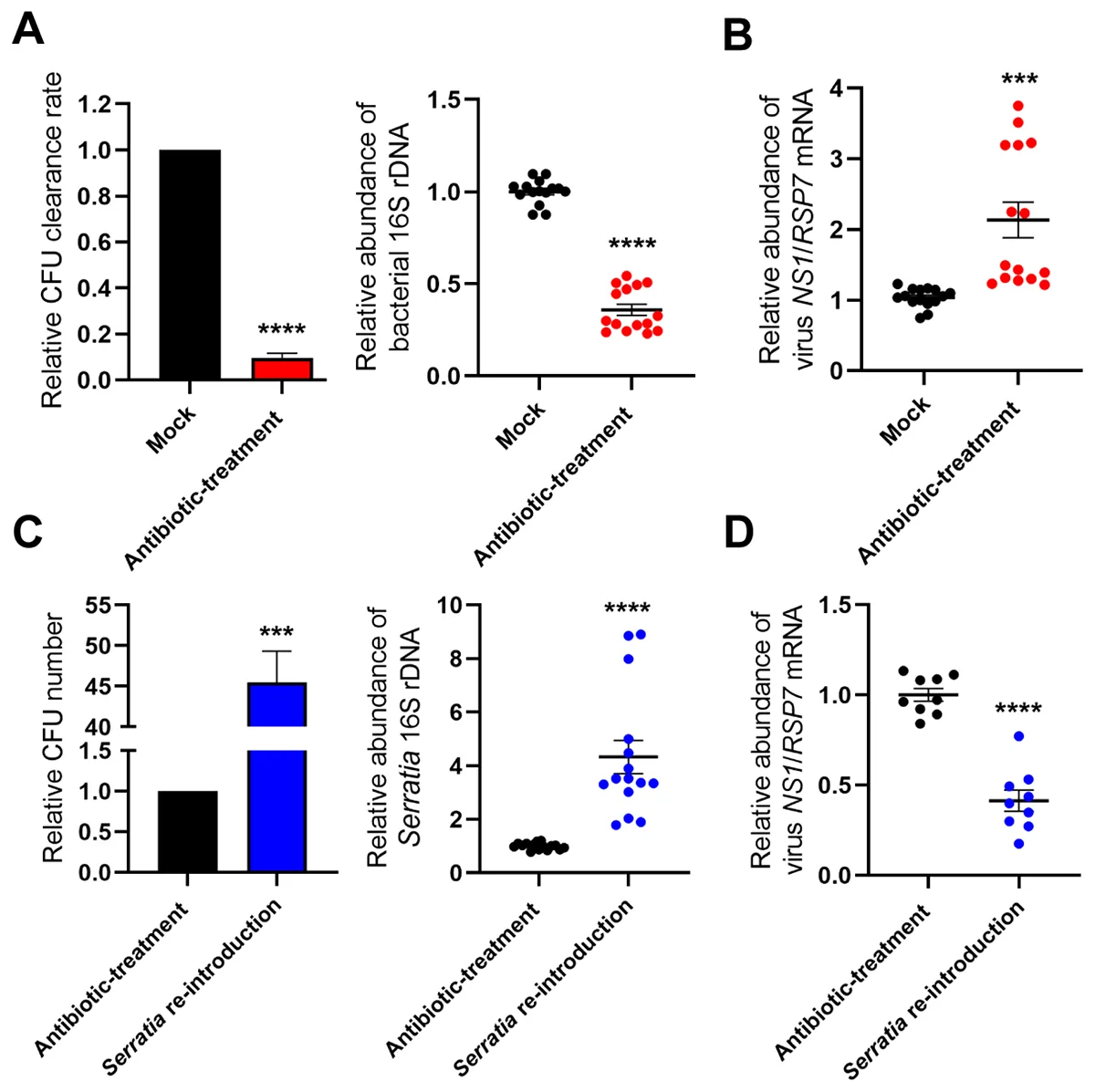
Colonization of *Ae. albopictus* by *S. marcescens* WZ1 inhibits DENV infection. (A) Assessment of gut microbiota clearance efficiency after antibiotic treatment. Midguts from *Ae. albopictus* fed with an antibiotic-containing sucrose solution for 5 days were homogenized, and bacterial load was evaluated by diluted CFU on agar plates and quantified via qPCR targeting bacterial 16S rDNA. (B) Effect of gut microbiota depletion on DENV susceptibility. Antibiotic-treated mosquitoes were infected with DENV2 via blood meal, and viral *NS1* RNA levels in midguts were measured by RT-qPCR at 3 days post-infection. (C) Evaluation of *S. marcescens* WZ1 recolonization efficiency. After feeding mosquitoes with a sucrose solution containing *S. marcescens* WZ1 for 3 days, bacterial colonization was assessed by colony counting and qPCR targeting the *Serratia*-specific 16S rDNA. (D) Impact of *S. marcescens* WZ1 recolonization on DENV susceptibility in mosquitoes. Antibiotic-treated mosquitoes recolonized with *S. marcescens* WZ1 were infected with DENV2 via blood meal. Viral *NS1* RNA levels in midguts were analyzed by RT-qPCR at 3 days post-infection. For 2A and 2C, CFU-based analyses are presented as relative values after pooling and normalization to the corresponding control pools, as described in the Methods, whereas qPCR-based bacterial abundance was analyzed from individual mosquito midguts. For 2B and 2D, each data point represents one pooled sample consisting of three dissected midguts. Data were pooled results from two independent experiments and are shown as mean ± SEM. Unpaired t test was used to determine the significance (***p < 0.001, ****p < 0.0001).

To validate the antiviral role of *S. marcescens* WZ1 *in vivo*, we first cleared the native gut microbiota of laboratory-reared *Ae. albopictus* using an antibiotic cocktail administered in sucrose solution. After 5 days of treatment, viable bacterial counts in mosquito midguts decreased by over 90% (Figure 2(A), left), and qPCR quantification of bacterial 16S rDNA confirmed a 64.1% reduction in total bacterial abundance (*p* < 0.0001, Figure 2(A), right), indicating effective gut microbiota depletion. We then examined the effect of microbiota depletion on DENV susceptibility. When antibiotic-treated mosquitoes were infected with DENV2 via blood meal, viral RNA copies in midguts increased by 133.3% compared to untreated controls (*p* < 0.001, Figure 2(B)), suggesting that the native microbiota exerts a basal protective effect against DENV infection.

To assess the protective capacity of *S. marcescens* WZ1, we recolonized the strain into antibiotic-treated mosquitoes via bacterial-supplemented sucrose feeding. This resulted in significant colonization, as evidenced by a 4.3-fold increase in *Serratia*-specific 16S rDNA gene copies compared to the control group (Figure 2). Importantly, mosquitoes colonized with WZ1 exhibited significantly reduced DENV2 infection intensity, showing a 58.7% decrease in viral RNA levels (*p* < 0.0001, Figure 2). These results demonstrate that specific colonization with *S. marcescens* WZ1 enhances mosquito resistance to DENV infection.

### *S. marcescens* WZ1-derived prodigiosin inhibits intracellular DENV proliferation

**Figure 3.**
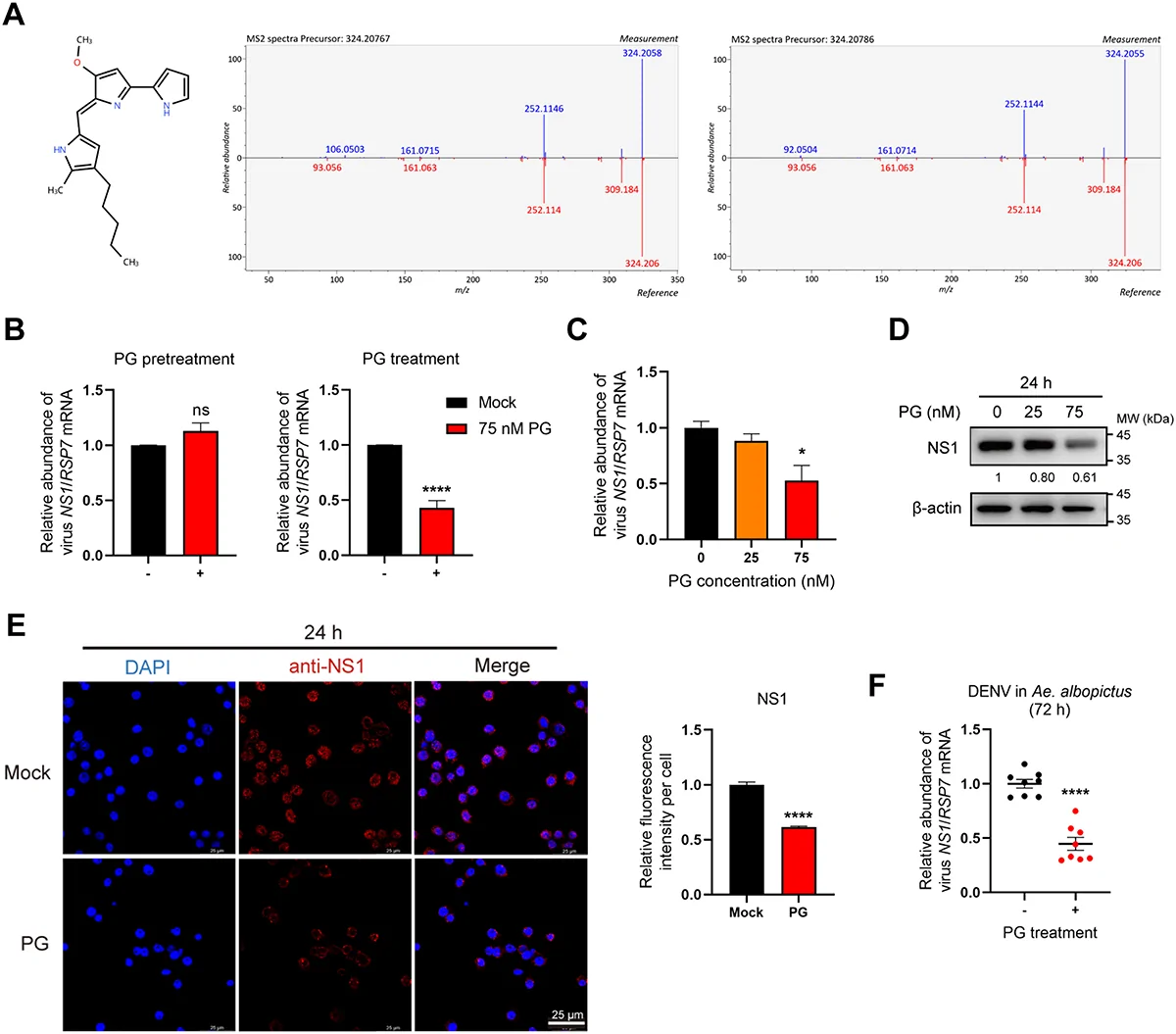
*S. marcescens* WZ1-derived prodigiosin acts as an antiviral metabolism *in vitro* and *in vivo*. (A) Identification of prodigiosin (PG) from *S. marcescens* WZ1 by HPLC-MS/MS. Left: chemical structure of PG. Middle and right: mass-to-charge (m/z) ratios of PG from the WZ1 strain and a commercial standard respectively (blue); signals in red are derived from database matches. (B) Anti-DENV activity of PG *in vitro*. C6/36 cells were treated with PG 2 h before (left) or after (right) DENV2 infection. Intracellular *NS1* RNA levels were measured by RT-qPCR at 48 h post-infection. (C, D) Dose-dependent inhibition of DENV infection by PG. Viral load in C6/36 cells treated with 25 nM or 75 nM PG for 24 h post-DENV2 infection was assessed by RT-qPCR (C) and western blot (D). (E) Immunofluorescence analysis of DENV2 NS1 expression in cells after PG treatment. Representative images (left) exemplify viral NS1 (red) and nuclei (blue). Mean fluorescence intensity (MFI) of NS1 per cell was quantified (right). (F) Anti-DENV effect of PG in mosquitoes. *Ae. albopictus* mosquitoes were fed a blood meal containing DENV2 mixed 1:1 (v/v) with 5 µM PG. DENV2 RNA levels in mosquito midguts were analyzed at 72 h post-infection by RT-qPCR. Each data point represents one pooled sample consisting of three dissected mosquito midguts. Data were representative or pooled results from two to three independent experiments and are shown as mean ± SEM. Ordinary one-way ANOVA with Dunnett’s multiple comparisons test (C) or unpaired t test (B, E, and F) were used to determine the significance (*p < 0.05, ****p < 0.0001, ns, not significant).

To identify the antiviral components secreted by *S. marcescens* WZ1, we performed metabolomic profiling of the bacterial supernatant using HPLC-MS/MS. Among the detected metabolites, prodigiosin (PG) was identified as one of the most abundant compounds. Mass spectrometry analysis confirmed that the structure of this metabolite was highly consistent with that of a commercially available PG standard (Abcam, ab144331; Figure 3(A)). Therefore, the compound was used as a surrogate for further investigation into the anti-DENV mechanism of *S. marcescens* WZ1. Given previous reports of PG’s antiviral activity against BmNPV and HSV [16,17], we selected this metabolite for further mechanistic studies.

We first established the non-cytotoxic concentration range of PG in C6/36 cells (Figure S1(A)) and then evaluated its anti-DENV activity at different infection stages. Interestingly, PG pretreatment before viral adsorption showed no significant effect, whereas administration after virus entry resulted in a 57.2% reduction in intracellular DENV2 RNA levels (*p* < 0.0001, Figure 3(B)), indicating that PG acts primarily on intracellular viral infection. Furthermore, PG demonstrated dose-dependent inhibition of DENV2 infection at both 24 and 48 h post-infection, as evidenced by decreasing *NS1* RNA levels and protein expression (Figure 3(C,D), Figure S1(B)). Immunofluorescence analysis confirmed these findings, showing 38.6% (*p* < 0.0001) and 50.7% (*p* < 0.01) reductions in NS1 fluorescence intensity at 24 and 48 h, respectively (Figure 3(E), Figure S1(C)). To assess the generality of the antiviral effect of PG, we also examined its activity against DENV2 during the intracellular stage in Aag2 cells. Consistent with the results in C6/36 cells, PG treatment significantly reduced intracellular DENV2 NS1 protein levels in Aag2 cells (Figure S1(D)). To evaluate the antiviral activity of PG in mosquitoes, we provided *Ae. albopictus* with a blood meal containing DENV2 mixed with 5 μM PG. At 3 days post-infection, RT-qPCR analysis showed a 54.6% reduction in midgut DENV2 RNA levels compared with the control group (*p* < 0.0001; Figure 3(F)). Together, these results demonstrate that PG exerts anti-DENV activity not only in cell culture but also in the complex physiological environment of the mosquito midgut, where it significantly reduces DENV2 RNA levels.

### Prodigiosin activates cellular autophagy to inhibit DENV infection

**Figure 4.**
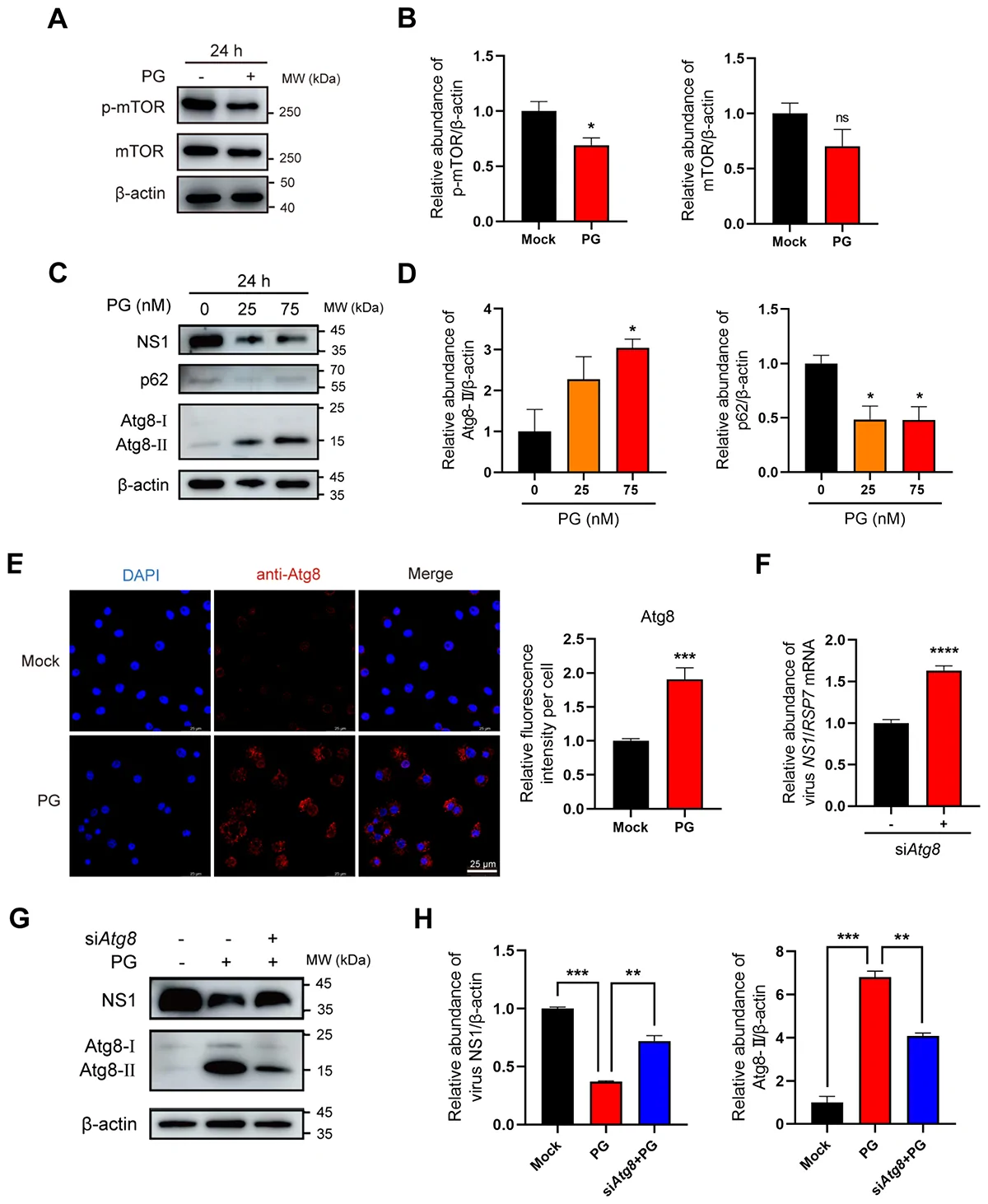
Prodigiosin activates autophagic flux to inhibit DENV infection. (A–D) PG suppresses the mTOR signaling pathway and induces autophagy. (A, B) Expression levels of total mTOR and phospho-mTOR in C6/36 cells treated with 75 nM PG were analyzed by western blot. (C, D) Expression levels of the autophagosome marker Atg8 and the autophagic flux indicator SQSTM1/p62 in DENV2-infected C6/36 cells treated with increasing concentrations of PG were quantified by western blot. (E) Increased Atg8 immunofluorescence signals after 24 h PG treatment. Representative images (left) show Atg8 (red) and nuclei (blue), and MFI of Atg8 per cell was quantified (right). (F–H) *Atg8* knockdown reverses the antiviral effect of PG. C6/36 cells were transfected with si*Atg8* prior to PG treatment. mRNA and protein levels of *Atg8* and DENV2 *NS1* were assessed by RT-qPCR (F) and western blot (G, H), respectively. Data were representative or pooled results from two to three independent experiments and are shown as mean ± SEM. Ordinary one-way ANOVA with Dunnett’s multiple comparisons test (D and H) or unpaired t test (B, E, and F) were used to determine the significance (*p < 0.05, **p < 0.01, ***p < 0.001, ****p < 0.0001, ns, not significant).

Previous studies have established that DENV hijacks the PI3K/AKT/mTOR signaling pathway to facilitate its replication [25], while this signaling cascade also serves as a central regulator of autophagy in insect cells. Interestingly, PG has been recently recognized as a potential autophagy inducer in cancer therapy [26,27]. We therefore investigated whether PG modulates autophagy-related signaling in mosquito cells. In PG-treated C6/36 cells, we observed significant downregulation of phospho-mTOR levels by 30.9% (*p* < 0.05; Figure 4(A,B)). Concurrently, PG treatment promoted the lipidation of Atg8, as evidenced by dose-dependent increases in the Atg8-II ratio, with 75 nM PG elevating Atg8-II levels by 3.04-fold (*p* < 0.05; Figure 4(C,D)). The autophagic substrate SQSTM1/p62 showed no accumulation, suggesting unimpaired autophagic flux. These trends were consistently observed at 48 h post-infection (Figure S2(A)). Immunofluorescence analysis further confirmed autophagy induction, showing a 1.91-fold increase in Atg8 signal intensity in PG-treated cells (*p* < 0.001, Figure 4(E)).

To determine whether PG suppresses DENV proliferation through enhanced autophagy, we employed RNA interference to knockdown *Atg8* expression prior to DENV2 infection. Transfection with si*Atg8* significantly reduced both mRNA and protein levels of Atg8 (including Atg8-I and Atg8-II) regardless of PG treatment (Figure S2(B,C)). Notably, *Atg8* knockdown substantially reversed the antiviral effect of PG, resulting in a 63.2% increase in DENV2 RNA copies (*p* < 0.0001; Figure 4(F)). Similarly, viral NS1 protein levels—which decreased by 63.1% (*p* < 0.001) with PG treatment—rebounded by 95.1% (*p* < 0.01) upon additional si*Atg8* transfection (Figure 4(G,H)). Throughout these experiments, Atg8-II expression exhibited an inverse correlation with DENV2 proliferation. Collectively, these data demonstrate that PG exerts its intracellular anti-DENV activity primarily through activation of cellular autophagy.

### Prodigiosin induces endoplasmic reticulum stress to activate autophagy

**Figure 5.**
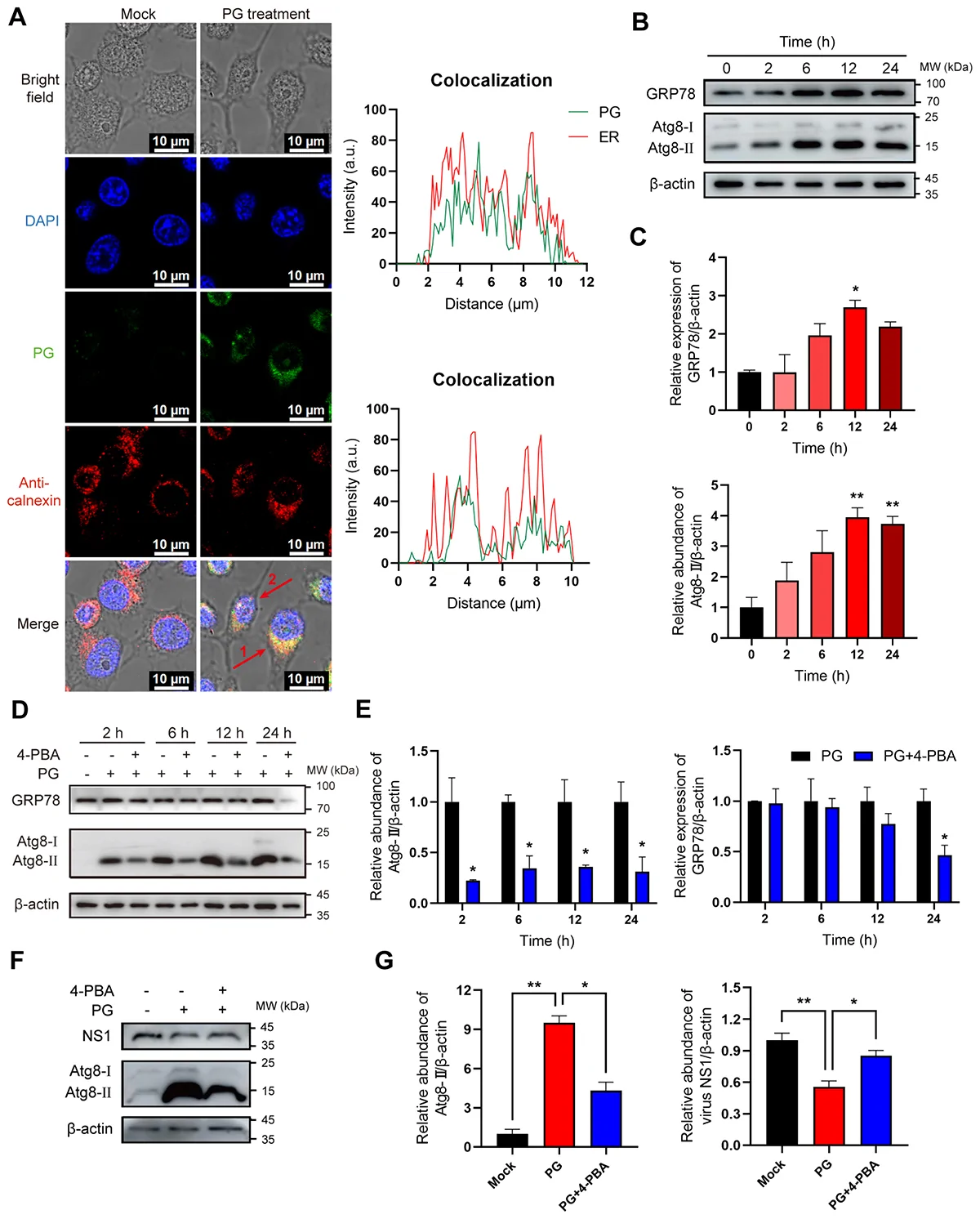
Prodigiosin induces autophagy by mediating endoplasmic reticulum stress. (A) Immunofluorescence microscopy reveals the colocalization of PG with the endoplasmic reticulum (ER). Representative images show PG (green) and calnexin-labeled ER (red) signals. Colocalization of two signals in cell 1 (above) and cell 2 (below) were analyzed using the Plot Profile in ImageJ. (B, C) PG treatment activates ER stress in *Ae. albopictus* cells. C6/36 cells were treated with 75 nM PG for 0–24 h. Expression levels of GRP78 and Atg8 were detected by western blot (B) and normalized to the untreated group (C). (D, E) Inhibition of ER stress attenuates PG-induced autophagy. ER stress was suppressed with 1 mM 4-PBA 2 h before PG addition. Protein levels of Atg8-II and GRP78 were quantified at the indicated time points. (F, G) inhibition of ER stress impairs the anti-DENV effect of PG. C6/36 cells were pretreated with 1 mM 4-PBA 2 h before DENV2 infection, followed by 48 h culture with PG. Expression of Atg8 and DENV2 NS1 was analyzed by western blot (F), and viral load was quantified taking PG-only and untreated groups as controls (G). Data were representative or pooled results from two to three independent experiments and are shown as mean ± SEM. Ordinary one-way ANOVA with Dunnett’s multiple comparisons test (C and G) or two-way ANOVA with Sidak’s multiple comparisons test (E) were used to determine the significance (*p < 0.05, **p < 0.01).

Subcellular localization analysis revealed significant accumulation of PG in the endoplasmic reticulum (ER) of C6/36 cells, as demonstrated by strong colocalization with the ER marker calnexin (Figure 5(A)). This prompted us to investigate whether PG triggers ER stress—an important upstream inducer of autophagy [28,29]. Time-course analysis showed that glucose-regulated protein 78 (GRP78), a central ER chaperone, began to increase as early as 2 h post-PG treatment and reached 2.7-fold higher levels than controls by 12 h (*p* < 0.05; Figure 5(B,C)). This upregulation coincided with elevated Atg8-II expression, suggesting a correlation between ER stress induction and autophagy activation.

To determine the functional relationship between PG-induced ER stress and autophagy, we inhibited ER stress using 4-phenylbutyric acid (4-PBA). Pretreatment with 1 mM 4-PBA, confirmed to be non-cytotoxic and effective in suppressing GRP78 expression (Figure S3(A,B)), significantly attenuated PG-induced autophagy, reducing Atg8-II levels by 60–80% (*p* < 0.05; Figure 5(D,E)). In contrast, transfection with si*Atg8* to suppress autophagy significantly reduced Atg8-II levels but had no effect on GRP78 expression (Figure S3(C,D)), indicating that ER stress acts upstream of autophagy without feedback regulation. Importantly, inhibition of ER stress also compromised the antiviral activity of PG, leading to a 53.3% rebound in intracellular NS1 protein levels (*p* < 0.05; Figure 5(F,G)). These results demonstrate that PG induces autophagy through the preceding activation of ER stress, thereby restricting DENV proliferation.

### Prodigiosin promotes autolysosome formation to clear DENV

**Figure 6.**
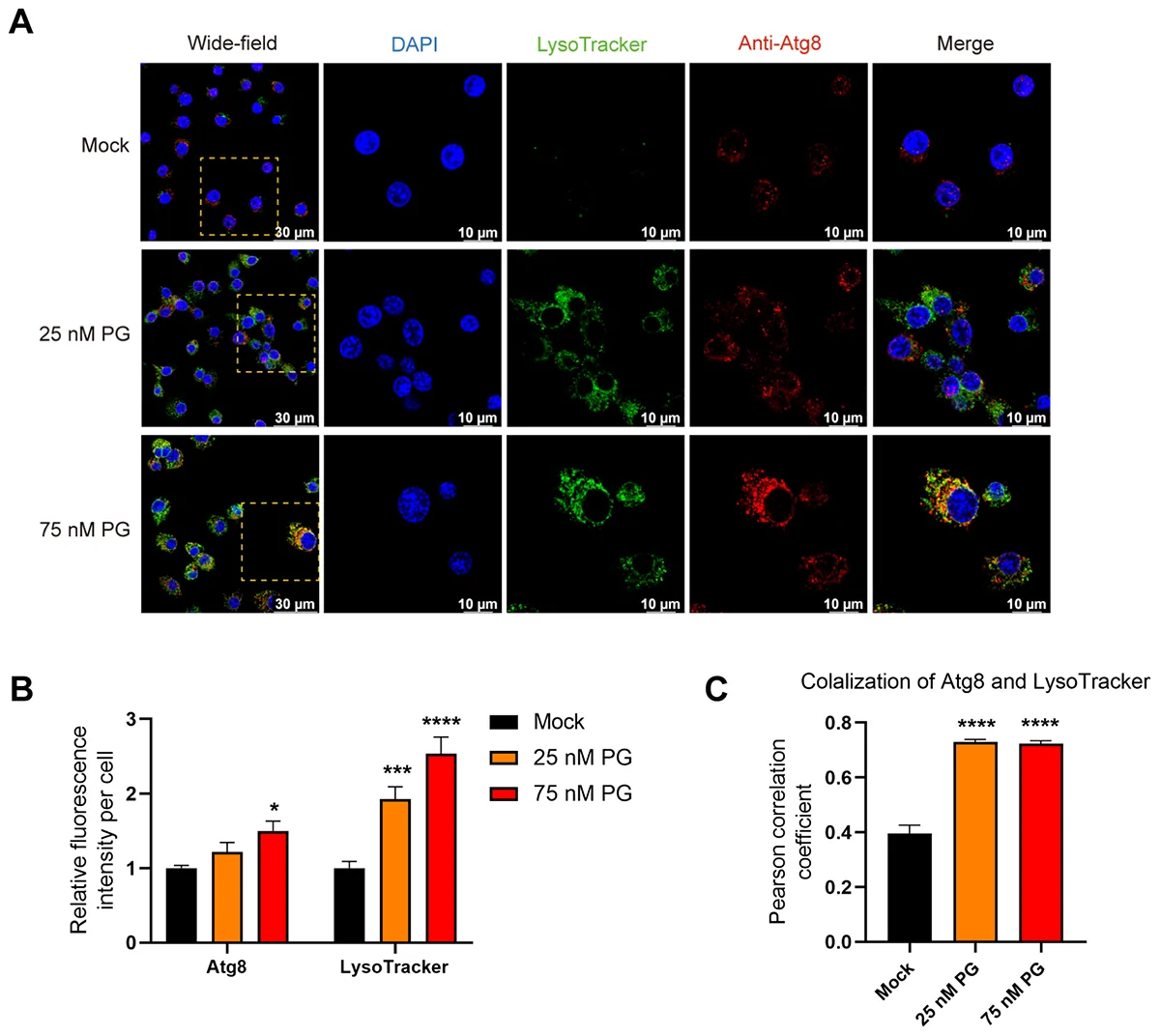
Prodigiosin promotes the formation of autolysosomes in *Aedes albopictus* cells. (A, B) PG upregulates lysosomal and autophagosomal signals in mosquito cells. (A) Representative immunofluorescence microscopy images show LysoTracker-labeled lysosomes (green) and Atg8-labeled autophagosomes (red) in C6/36 cells treated with increasing concentrations of PG. (B) the corresponding signals after 24 h incubation with 25 nM or 75 nM PG were quantified as MFI per cell. (C) PG treatment significantly enhances colocalization of lysosomes with autophagosomes. Pearson correlation coefficient (PCC) between LysoTracker and Atg8 signals was calculated using ImageJ across at least six random fields. Data were representative or pooled results from two independent experiments and are shown as mean ± SEM. Ordinary one-way ANOVA with Dunnett’s multiple comparisons test (C) or two-way ANOVA with Sidak’s multiple comparisons test (B) were used to determine the significance (*p < 0.05, ***p < 0.001, ****p < 0.0001).

To elucidate how PG-activated autophagy reduces intracellular DENV particles, we investigated the formation of autolysosomes – the functional organelles responsible for pathogen degradation. Immunofluorescence analysis revealed that PG treatment dose-dependently enhanced the signals of both the autophagosome marker Atg8 and lysosomal compartments in DENV2-infected C6/36 cells at 24 h post-treatment (Figure 6(A)). Quantification showed that lysosomal signals increased by 1.92-fold (*p* < 0.001) and 2.53-fold (*p* < 0.0001) after treatment with 25 nM and 75 nM PG, respectively (Figure 6(B)). Critically, Pearson correlation coefficient (PCC) analysis demonstrated significantly enhanced colocalization between autophagosomes and lysosomes in PG-treated groups (Control: 0.395; 25 nM PG: 0.730; 75 nM PG: 0.723; *p* < 0.0001; Figure 6), indicating robust autolysosome formation. This phenomenon was further confirmed at the earlier 12 h time point (Figure S4(A-C)), suggesting rapid and sustained autolysosome formation.

**Figure 7.**
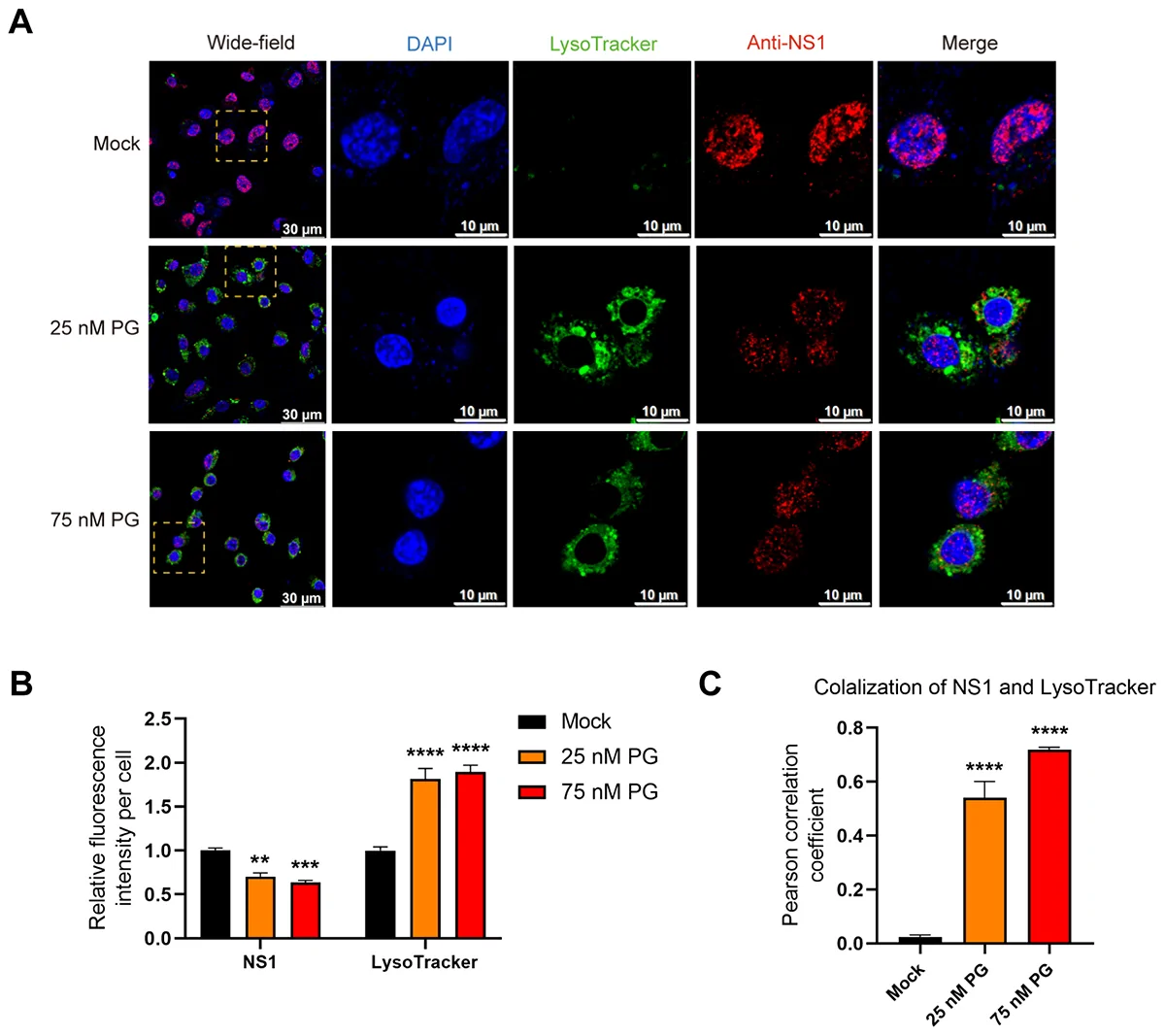
Prodigiosin-induced autolysosomes mediate clearance of DENV. (A, B) PG increases autolysosome formation while reducing intracellular viral levels. (A) Representative immunofluorescence images show LysoTracker-labeled lysosomes/autolysosomes (green) and NS1-labeled DENV2 (red) in C6/36 cells treated with increasing concentrations of PG. (B) MFI per cell was quantified in DENV2-infected cells treated with 25 nM or 75 nM PG for 48 h. (C) PG significantly enhances colocalization between lysosomes/autolysosomes and DENV2. PCC between LysoTracker and NS1 signals was calculated using ImageJ across at least six random fields. Data were representative or pooled results from two independent experiments and are shown as mean ± SEM. Ordinary one-way ANOVA with Dunnett’s multiple comparisons test (C) or two-way ANOVA with Sidak’s multiple comparisons test (B) were used to determine the significance (**p < 0.01, ***p < 0.001, ****p < 0.0001).

We next examined whether PG-induced autolysosomes physically interact with virions. Immunofluorescence tracking showed that increasing PG concentrations progressively reduced NS1 fluorescence intensity by 29.8% (*p* < 0.01) with 25 nM PG and 36.4% (*p* < 0.001) with 75 nM PG (Figure 7(A,B)). More importantly, colocalization analysis revealed that while the PCC between LysoTracker and DENV2 signals was minimal in untreated cells (0.023) at 48 h post-infection, it dramatically increased to 0.540 (*p* < 0.0001) and 0.718 (*p* < 0.0001) with 25 nM and 75 nM PG treatment, respectively (Figure 7). Similar enhancement in virus-lysosome/autolysosome colocalization was observed at 24 h post-infection (Figure S4(D-F)). These data provide direct visual evidence that PG promotes the sequestration of DENV particles into autolysosomes, leading to their subsequent degradation.

## Discussion

**Figure 8.**
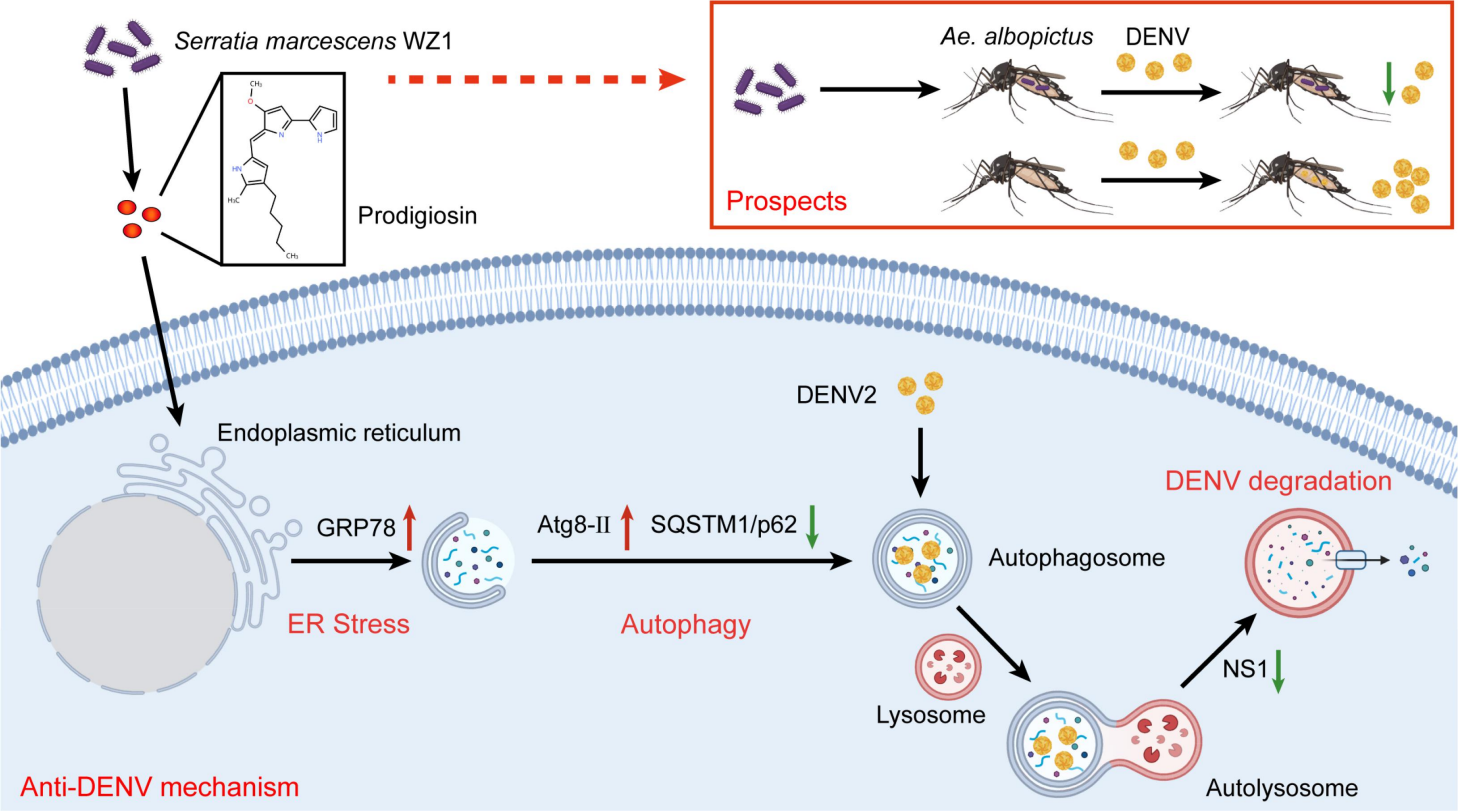
Antiviral autophagy induced by prodigiosin: mechanism and translational potential of *Serratia marcescens* WZ1 against DENV.

In this study, we identified *Serratia marcescens* WZ1, a gut commensal bacterium isolated from field-caught *Ae. albopictus* in Wenzhou, China, which exhibits inhibitory activity against DENV infection. Employing integrated *in vitro* and *in vivo* approaches, we demonstrated that its active metabolite, prodigiosin (PG), significantly suppresses viral replication in both mosquito cells and mosquito midguts. Mechanistically, we established that PG triggers endoplasmic reticulum (ER) stress, leading to the induction of a complete autophagic flux that culminates in the degradation of virions via enhanced autolysosome formation (Figure 8). Although other WZ1-derived secreted factors may also contribute to the overall antiviral activity of the bacterial supernatant, this does not diminish the significance of PG as a functional metabolite from a natural mosquito gut symbiont that suppresses arboviral infection by activating a host degradative pathway.

The gut microbiota plays a pivotal role in shaping mosquito vector competence for arboviruses, with different symbiotic bacteria exhibiting divergent influences on viral infection. For instance, while *Serratia odorifera* has been reported to facilitate DENV infection [30], others like *Chromobacterium* species can suppress it through secreted aminopeptidases that degrade viral envelopes [13]. Our discovery that *S. marcescens* WZ1 secretes PG to exert potent antiviral activity adds a layer of functional complexity to the *Serratia* genus. This is particularly striking when contrasted with another *S. marcescens* strain that enhances arboviral infection via the secretion of SmEnhancin, which disrupts the midgut epithelial barrier [11]. Such functional dichotomy among different strains of the same species highlights the critical importance of strain-level functional variation in determining microbial influence on viral susceptibility [31], the specificity of which may arise from variations in genetic background, metabolic profiles, and interactions with the host immune system. This concept resonates with findings in human gut microbiome research, where distinct subclades of *Faecalibacterium prausnitzii* exhibit specialized metabolic functions in patients with liver cirrhosis [32], challenging the conventional species-level generalizations. Our work thus underscores that strain-specific metabolic capabilities, particularly the production of metabolites such as PG ultimately determines bacterial impact on host-pathogen interactions.

Autophagy represents a conserved cellular degradation pathway that typically functions in host defense by eliminating intracellular pathogens. However, DENV has evolved to exploit this pathway through a sophisticated dual strategy: it exploits early autophagosomes as protected scaffolds for its replication machinery, shielding from host antiviral factors and concentrating the necessary components for efficient genome replication [33–35], while simultaneously interfering with the later stages of the autolysosome formation to prevent the degradation of viral components [36]. This manipulation results in an incomplete autophagic flux, characterized by the accumulation of LC3-II alongside elevated levels of the autophagy substrate SQSTM1/p62 [37]. Moreover, the DENV non-structural protein NS1 contributes to this blockade by binding to Beclin-1 and impeding its caspase-mediated cleavage—an event that would otherwise promote autophagic termination and apoptosis [37,38]. Crucially, our findings demonstrate that PG effectively counteracts this viral hijacking by restoring complete autophagic flux, ultimately directing virions to degradation within autolysosomes. This “reversal” strategy represents a novel antiviral approach: instead of directly targeting viral components, PG reclaims control over the host pathway co-opted by DENV, thereby repurposing the virus’s own survival mechanism against itself.

While PG has been extensively studied for its anticancer, antibacterial, and immunosuppressive properties [39], its antiviral functions remain comparatively underexplored, particularly in mosquito–arbovirus systems. Intriguingly, existing literature reveals cell type-specific differences in how PG modulates autophagy. In glioblastoma and corneal epithelial cells, PG inhibits the AKT/mTOR pathway and consistently promotes autophagic activation [40,41], aligning with our observations in DENV-infected mosquito cells. In contrast, PG treatment in HeLa cells results in blocked autophagic flux, as indicated by the concurrent accumulation of LC3-II and SQSTM1/p62 [42]. These divergent outcomes underscore the context-dependent nature of PG’s bioactivity and highlight the necessity of evaluating its functions within biologically relevant host–pathogen systems. Our work establishes a mosquito cell model where PG induces a complete and functional autophagic response, effectively countering DENV infection.

The subcellular localization of PG provides crucial insights into its functional mechanism. Our immunofluorescence tracking revealed significant accumulation of PG in the endoplasmic reticulum (ER) of C6/36 cells, where it induces ER stress and upregulates GRP78 expression. This ER-targeting behavior aligns with previous reports of PG-induced ER stress in other systems [40]. Notably, although PG has been shown to bind GRASP55 and disrupt autophagosome-lysosome fusion in HeLa cells [42], we observed complete autophagic flux with enhanced viral degradation in autolysosomes. Our findings reveal that PG exploits ER stress to activate a pro-degradative autophagic program, effectively repurposing a cellular stress response into an antiviral defense. Given that DENV replication depends on ER-derived membrane structures [43], ER stress/unfolded protein response (UPR) modulation may affect DENV production in a time- and branch-dependent manner. For example, pharmacological modulation of eIF2α dephosphorylation by Salubrinal significantly reduced DENV2 production [44]. We therefore interpret PG-induced GRP78 upregulation as a marker of ER stress that may render the ER-associated replication environment unfavorable and redirect viral components toward autophagic–lysosomal degradation, rather than as evidence that PG physically disrupts replication factories. Further studies will be required to resolve both the specific UPR branch linking PG-induced ER stress to autophagy and its impact on ER-derived DENV replication compartments.

The anti-DENV activity of *S. marcescens* WZ1 and its metabolite PG provides candidate biological tools and a mechanistic basis for developing novel transmission-blocking interventions. Based on our findings, two potential directions may be considered in subsequent work (Figure 8): a microbiome-based approach, in which WZ1 could be deployed as a probiotic symbiont to render mosquito populations refractory to viral infection—analogous to *Wolbachia*-based population replacement [45,46]; and a metabolite-based strategy, wherein PG or its derivatives could be formulated into sugar baits or larvicides to directly suppress viral replication in field settings.

Although previous work has reported stage- and context-dependent mosquito toxicity of *S. marcescens* secondary metabolites, particularly in larvae [47], no obvious increase in adult mosquito mortality was observed under the WZ1 recolonization or PG feeding conditions used here. Nevertheless, systematic evaluation of long-term adult mosquito survival and fitness will be necessary before WZ1- or PG-based strategies can be further developed. In addition, although PG has been shown to reduce DENV-associated markers in the mosquito midgut during early infection, viral dissemination within mosquitoes and subsequent transmission potential still require dedicated investigation. The successful deployment of *Rosenbergiella* sp. YN46 in semi-field conditions via introduction into breeding water offers an encouraging precedent, yet also underscores the need for context-specific optimization [48]. Similarly, the pharmacokinetics, delivery formulations, and field stability of PG require systematic development to enable its effective application. Finally, evaluating the broad-spectrum efficacy of this approach against other medically relevant arboviruses, such as ZIKV and chikungunya viruses, also represents an important direction for future research.

## Conclusions

This study identifies the mosquito gut symbiont *Serratia marcescens* WZ1 and its functional metabolite prodigiosin as inhibitors of DENV infection. We further delineate an antiviral mechanism in which PG induces ER stress and activates a functional autophagic flux, promoting lysosomal targeting and clearance of DENV-associated components. Collectively, these findings deepen our understanding of mosquito–microbe–virus interactions and provide a mechanistic framework and candidate leads for future studies aimed at reducing arboviral infection in mosquito vectors.

## Supplementary Material

Supplemental Material

## Data Availability

The data that support the findings of this study are openly available at https://doi.org/10.6084/m9.figshare.30674600 .
